# Homo-β-amino acid containing MBP(85–99) analogs alleviate experimental autoimmune encephalomyelitis

**DOI:** 10.1038/srep08205

**Published:** 2015-02-03

**Authors:** Ravi Kant, Shweta Pasi, Avadhesha Surolia

**Affiliations:** 1 Molecular Science Laboratory, National Institute of Immunology, New Delhi-110067, India

## Abstract

MBP(85–99), an immuno-dominant epitope of myelin basic protein which binds to the major histocompatibility complex haplotype HLA-DR2 is widely implicated in the pathogenesis of multiple sclerosis. J5, an antagonist of MBP(85–99), that blocks the binding of MBP(85–99) to soluble HLA-DR2b much more efficiently than glatiramer acetate (a random copolymer comprising major MHC and T-cell receptor contact residues), was transformed into analogs with superior biological half-lives and antagonistic-activities by substitution of some of its residues with homo-β-amino acids. S18, the best analog obtained ameliorated symptoms of experimental autoimmune encephalomyelitis at least twice more effectively than glatiramer acetate or J5. S18 displayed marked resistance to proteolysis *in-vitro*; biological impact of which was evident in the form of delayed clinical onset of disease and prolonged therapeutic-benefits. Besides active suppression of MBP(85–99)-reactive CD4^+^ T-cells *in-vitro* and *in-vivo* S18 treatment also generated IL-4 producing CD4^+^ T-cell clones, through which protective effect could be transferred passively.

Multiple Sclerosis (MS), a chronic autoimmune demyelinating disorder of central nervous system (CNS), is characterized by peripheral mononuclear infiltration, plaques in white matter and neurological dysfunction^1^. The disease usually starts in young adulthood; clinically, based on the symptomatic progression it has been classified into four types: benign, relapsing-remitting, secondary chronic progressive and primary progressive^2^. Of these four clinical phenotypes, relapsing-remitting MS (RRMS) is most common accounting for more than 85% of the cases followed by chronic progressive. MS has an overall prevalence of >2.5 million cases worldwide with high concentration in temperate climates^3^.

Epidemiological studies have revealed concurrence of MS with certain MHC haplotypes of which HLA-DR2 (HLA-DRB1*1501, HLA-DRB5*0101) has been mostly implicated^4^,^5^. Further, various constituents of the myelin sheath have been identified as target auto-antigens in the pathogenesis of MS^6^. These include myelin basic protein (MBP)^7^, proteolipid protein (PLP)^8^, myelin oligodendroglycoprotein (MOG)^9^, myelin associated glycoprotein (MAG)^10^, etc. In human subjects MBP(85–99) has been widely considered as an immuno-dominant epitope involved in the pathogenesis of MS^11^. Structural studies involving a tri-molecular complex of HLA-DR2b-MBP(85–99)-TCR have revealed valine (V), phenylalanine (F) at positions P1, P4 as major MHC anchors and histidine (H), phenylalanine (F), lysine (K), at P2, P3, P5 as TCR contact residues respectively^12^,^13^.

Pathological events in MS are believed to be either triggered by exposure to myelin cross-reactive antigens present on invading pathogens or the accidental release of myelin-components from CNS into the peripheral compartment. Subsequent to priming, the activated B and T lymphocytes egress from lymphoid tissue, migrate across the blood brain barrier (BBB) into the CNS where on re-encountering their cognate antigens they damage the myelin sheath^14^,^15^. Various components of pathogenic process have been targeted for designing therapeutic strategies such as: presentation of myelin antigens to auto-reactive T-cells, their activation, egress from lymph nodes, migration across vasculature and blood brain barrier^16^,^17^. Most of the therapeutics developed so far, suppress immune system non-specifically. Hence, development of antigen-specific therapies has always been of great interest.

Copolymer 1, glatiramer acetate (GA), an example of antigen-specific therapeutics, is currently the treatment of choice for RRMS. GA is a random copolymer of tyrosine (Y), glutamic acid (E), alanine (A), lysine (K), residues which constitutes major MHC anchors and TCR contacts (Molecular Weight 4.7 to 11 kDa, molar ratio 5:3:1:1.5)^18^,^19^. GA is known to exert its therapeutic benefit by blocking the priming of auto-reactive CD4^+^ T-cells^20^, shifting the Th1/Th2 balance towards Th2 (anti-inflammatory)^21^,^22^ and generation of myelin cross-reactive regulatory T-cells^23^.

However, therapeutic potencies of such antigen-specific modalities has always been moderate due to their peptidic nature e.g. in the case of GA it is only 20–30 percent^24^. Expectedly, it is their minimal stability in proteolytic milieu and thus inefficient presentation on antigen presenting cells (APCs) which perhaps gets translated into their reduced efficacy.

In the present study homo-β-amino acids have been employed to address the issue of limited biological half-life of MBP(85–99) antagonists such as J5^25^. Homo-β-amino acids have their amino group attached to β-carbon instead of α-carbon and their side chains (R groups) are identical to that of their naturally occurring counterparts. Certain residues in J5, the best known MBP(85–99) antagonist, were replaced with their homo-β-counterparts to obtain antagonists with far superior stability and antagonistic activities, features that eventually translated into 1.5 to 2 fold enhancement of therapeutic efficacy in experimental models of MS.

## Results

### Homo-β-amino acid substituted analogs of MBP(85–99): Design, synthesis and inhibition analysis

We initiated these studies with J5, one of the best known antagonist of MBP(85–99). The salient structural features of J5 in relation to its parent i.e. MBP(85–99) are summarized as follows. Firstly, phenylalanine (F) at position P4 has been replaced with tyrosine (Y), while valine (V) at position P1 has been retained. Secondly, histidine (H), phenylalanine (F), lysine (K) at P2, P3, P5 have been replaced with glutamic acid (E), alanine (A), lysine (K) respectively. Thirdly, the stretch of amino acids from position P6 to P11 has been replaced with alanines (As). Fourthly, amino acid residues at penultimate positions i.e. P10 and P-2 have been exchanged with prolines (Ps), since they are known for their ability to resist degradation by exo-peptidases (Supplementary Fig. S1). Stern et al. have demonstrated the ability of J5 to block the binding of MBP(85–99) to soluble HLA-DR2b twice more effectively than GA^25^. However, the therapeutic efficacy of J5 is limited like any other peptide therapeutic which may be attributed to its low biological half-life or bioavailability. This limitation of biological half-life in the present case was addressed by a systematic replacement of certain residues in J5 with their homo-β counterparts in a manner such that its inhibitory activity is not compromised.

**Figure 1:**
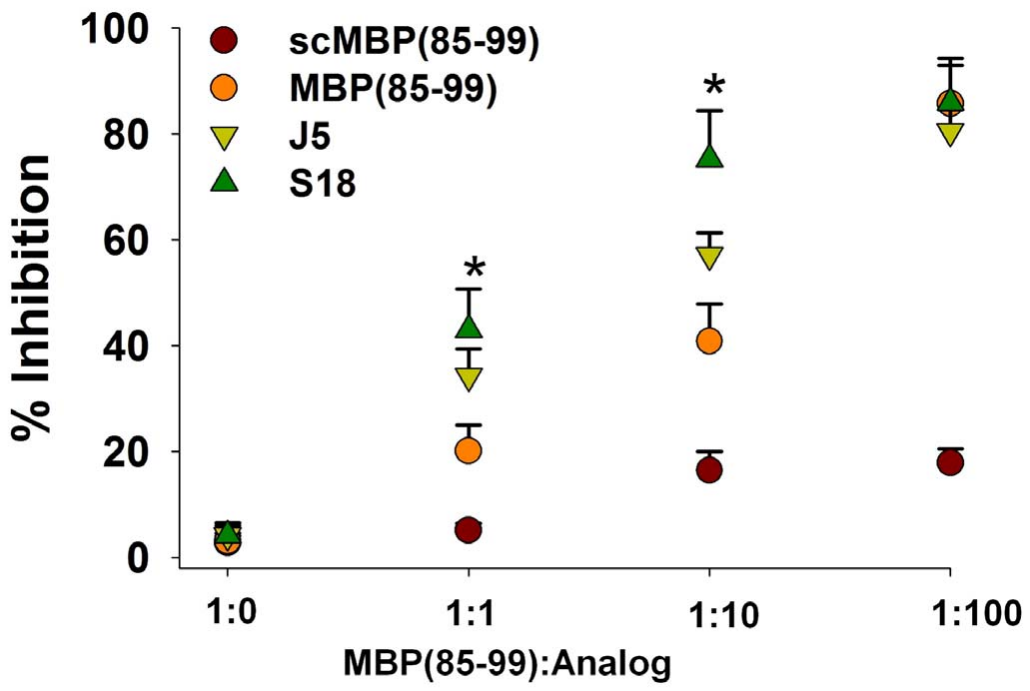
S18 antagonizes binding of MBP(85–99) to soluble HLA-DR2b. Solubilized HLA-DR2b was incubated with biotin-MBP(85–99) and scrambled MBP(85–99) (control), MBP(85–99), J5 or S18 at various molar ratios. Percent inhibition. Mean ± S.D., *p<0.05, n ≥ 3, where n is number of independent experiments.

In the first set of analogs designed (S1 to S6) single substitutions were carried out in the region extending from position P6 to P11 as this stretch of amino acids is known to be less important for interaction with HLA-DR2b^12^,^26^. The inhibitory potentials of analogs designed were quantified in terms of their ability to block the binding of biotin-MBP(85–99) to soluble HLA-DR2b. Single substitutions in this region barely had any effect on their antagonistic function (Supplementary Table S1). In another set of analogs designed (S7 to S11) single replacements were carried out in the locus which harbors major MHC anchors and TCR contacts i.e. from position P1 to P5. In this case single substitutions at P1, P2, P3 or P4 were deleterious; the only possible substitution was that of lysine (K) at P5. All favorable substitutions obtained so far were thus accumulated into analog S12. Expectedly, inhibitory activity of analog S12 was almost comparable to that of J5 (Supplementary Table S1). Therefore, S12 was taken as a reference analog for further design. In yet another set of analogs designed (S13 to S16) possibility of further substitutions was explored. Unlike C-terminus only glutamic acid (E) and lysine (K) at P-4 (S15) and P-3 (S16) in N-terminal region could be replaced without compromising inhibitory activity (Supplementary Table S1). Therefore, all the favorable substitutions obtained so far were clubbed into analog S17. Blocking activity of S17 was found to be comparable to that of J5 (Supplementary Table S1). Thus antagonistic function could be retained while increasing β amino acid content (Supplementary Table S1). Further, considering the length of peptide which a class II MHC could accommodate in its groove, we looked for the possibility of truncations at C-terminus. The amino acid residues at C-terminus of MBP(85–99) when bound to HLA-DR2b are positioned higher and believed to be less important for interaction^12^,^26^. Thus truncated analog S18 derived from S17 (deleted P_β_^3^ and A_β_^3^ at P10 and P11) contains structural features important for binding to HLA-DR2b or contacting TCR. Surprisingly, S18 could antagonize the binding of MBP(85–99) much more efficiently. In fact, it was even better than J5 or MBP(85–99) itself (Supplementary Table S1). However attempts to further reduce the length to include major MHC anchors only i.e. analog S19, S20, were futile (Supplementary Table S1). The findings were further validated *in-silico* employing MOE software. The binding energies calculated for J5 and S18 were −9.8 ± 0.2 and −11.6 ± 0.3 respectively, which were significantly lower than that of MBP(85–99) (−7.1 ± 0.5) under identical experimental conditions. Besides, better binding affinities, the modes of binding for J5 and S18 were largely similar (Supplementary Fig. S2). Antagonistic function of S18, the best analog obtained was further analyzed in a greater detail. Inhibitory potentials of S18 were analyzed at various molar ratios. Obviously, the difference in antagonistic activity was even more pronounced at higher molar ratios. Also, the binding of MBP(85–99) to HLA-DR2b could be specifically blocked by MBP(85–99) only (Figure 1). To summarize, antagonistic activity of S18 was 10–15% greater than that of J5.

### S18 blocks binding of MBP(85–99) to cell surface HLA-DR2

**Figure 2:**
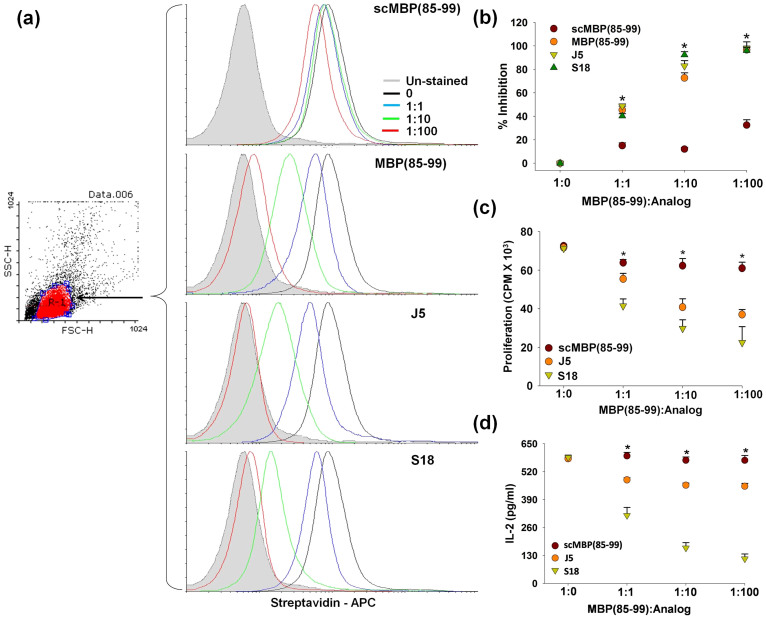
S18 blocks presentation of MBP(85–99) to CD4^+^ T-cells. MGAR cells growing logarithmically were irradiated and incubated with biotin-MBP(85–99) and/or scrambled MBP(85–99), MBP(85–99), J5 or S18. Biotin-MBP(85–99) bound to cell surface HLA-DR2 was estimated flowcytometrically. (a) Mean Fluorescence Intensity (MFI). (b) Percent inhibition. Irradiated MGAR cells pulsed with MBP(85–99) and scrambled MBP(85–99), J5 or S18 were co-cultured with Ob.1A12, (c) proliferative response and (d) levels of IL-2 in culture supernatants. Mean ± S.D., *p<0.05, n ≥ 3, where n is number of independent experiments.

The inhibitory potential of analog S18 was also quantified in terms of its ability to block the binding of biotin-MBP(85–99) to HLA-DR2 present on the surface of antigen presenting cells. Here, MGAR cells were employed as APCs. Irradiated MGAR cells were incubated with biotin-MBP(85–99) in the presence or absence of scrambled MBP(85–99), MBP(85–99), J5 or S18 and bound biotin-MBP(85–99) was detected using fluorochrome conjugated streptavidin. The binding of biotin-MBP(85–99), measured in terms of mean fluorescence intensity (MFI), exhibited two characteristic features; first, it followed a saturation kinetics with respect to biotin-MBP(85–99) concentration, second, it could be specifically blocked by MBP(85–99) only, which together demonstrate the specificity and sensitivity of the assay (Figure 2a). Expectedly, S18 antagonized the binding of MBP(85–99) to cell surface HLA-DR2 in concentration dependent fashion (Figure 2a–b) thereby, corroborating the findings of solution phase competition assay.

### S18 suppresses MBP(85–99) reactive CD4^+^ T-cell activation

In addition to its ability to competitively block the binding of MBP(85–99) to HLA-DR2b, an important aspect to be studied was its effect on activation of auto-reactive T-cells, which was analyzed in terms of its ability to suppress activation of MBP(85–99) reactive CD4^+^ T-cells *in-vitro*. Irradiated MGAR cells pulsed with MBP(85–99) and/or increasing concentrations of scrambled MBP(85–99), J5 or S18 were incubated with Ob.1A12 (an MS patient derived auto-reactive CD4^+^ T-cell clone which recognizes MBP(85–99) in the context of HLA-DR2b) and examined for proliferative activity and IL-2 production. The suppressive effect on T-cell function measured in terms of proliferation or IL-2 production was a lot more pronounced in the case of S18 than J5 which may be attributed to modifications of lysine (K), TCR contact at position P5 (Figure 2c–d). Hence, we concluded that analog S18 blocks the binding of MBP(85–99) to HLA-DR2b and at the same time does not activate auto-reactive T-cells.

### S18 is immunogenic but not cross-reactive to MBP(85–99)

**Figure 3:**
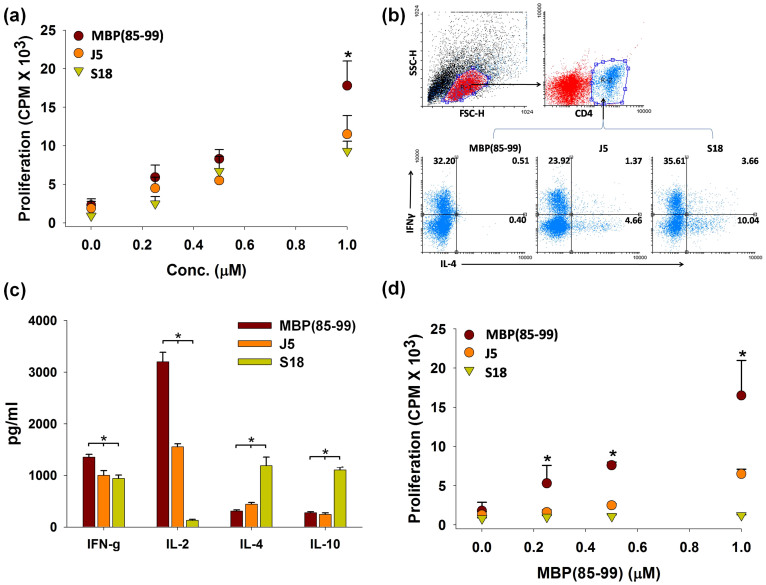
S18 is immunogenic but not cross-reactive to MBP(85–99). To evaluate immunogenicity *SJL/J* mice (8–10 week old, female) were immunized with 100 μg of MBP(85–99), J5 or S18 in complete Freund's adjuvant (CFA) subcutaneously and after 2 weeks splenocytes were monitored for proliferative response to (a) respective peptides. Splenocytes isolated were also analyzed flowcytometrically for Th1 (CD4^+^IFNγ^+^) and Th2 (CD4^+^IL-4^+^) phenotypes after repeated stimulation (every week) with respective peptides, (b) FACS dot plots and (c) levels of IFNγ, IL-2, IL-4, Il-10 in culture supernatants. (d) Proliferative activity of splenocytes derived from MBP(85–99), J5 or S18 immunized mice in response to MBP(85–99) treatment. Mean ± S.D., *p<0.05, n ≥ 3, where n is number of independent experiments.

*SJL/J* mice were injected with MBP(85–99), J5 or S18 emulsified in CFA. After two weeks splenocytes were isolated and proliferative response was quantified upon treatment with respective peptides. Besides MBP(85–99) both J5 and S18 were found to stimulate splenocytes, although to a lesser extent (Figure 3a). Subsequently, MBP(85–99), J5 or S18 reactive CD4^+^ T-cell clones obtained after repeated stimulation with respective peptides were analyzed for intracellular IL-4 and IFN-γ. The ratio of CD4^+^ T-cells exhibiting Th1 (CD4^+^ IFN-γ^+^) or Th2 (CD4^+^ IL-4^+^) phenotype (Th1:Th2) observed were 80.5, 5.13 and 3.54 in MBP(85–99), J5 and S18 respectively. Therefore, contrary to MBP(85–99), immunological response to S18 was predominantly of Th2 type (Figure 3b). Simultaneously, frequencies of Th1 (CD4^+^IFNγ^+^) and Th2 cells (CD4^+^IL-4^+^) correlated well with the levels of IL-4 and IFNγ observed in their supernatants (Figure 3c). These observations thus demonstrate immunogenicity and ability of S18 to polarize Th1-Th2 balance towards Th2, alternatively, generation of S18 reactive anti-inflammatory CD4^+^ T-cell clones. Further, a bare minimal proliferative activity in splenocytes derived from mice immunized with S18 upon *ex-vivo* stimulation with MBP(85–99) observed also suggested lack of cross-reactivity (Figure 3d).

### S18 is extremely resistant to proteolysis

**Figure 4:**
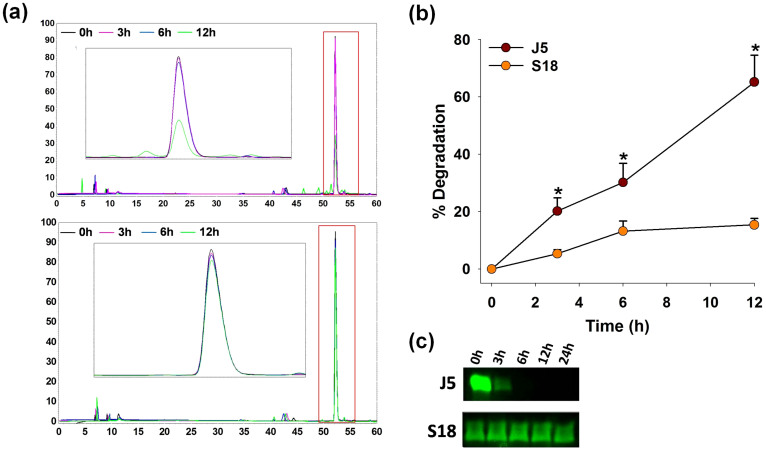
S18 is extremely resistant to proteolysis. J5 or S18 were incubated with α-chymotrypsin in assay buffer at 37°C, 50 μl aliquots were drawn at various time points and analyzed by reverse phase HPLC, (a) chromatograms, (b) percent degradation. To analyze stability in proteolytic environment FITC conjugated J5 or S18 were incubated in normal mouse serum, aliquots were drawn and electrophoresed, (c) PAGE (15%). Mean ± S.D., *p<0.05, n ≥ 3, where n is number of independent experiments.

To analyze the effect of homo-β-amino acid incorporation on proteolytic stability, S18 or J5 were incubated with chymotrypsin and analyzed at various time points. Beta-peptides are known for their remarkable resistance to a variety of proteolytic enzymes^27^. Chymotrypsin, a serine protease, was used to exemplify the same in the present case of a α-β hybrid peptide. Further, chymotrypsin was preferred as it selectively hydrolyzes peptide bonds on carboxy-terminus of hydrophobic amino acids (phenylalanine, tryptophan or tyrosine) and will thus cleave J5 or S18 at P5, the locus considered critical for their biological function. Analog S18 was found to be fiercely resistant to chymotrypsinization as >90% of it remained intact in reaction mixture even after 12 hours and was detectable in the reaction mixture upto 2 days. Contrary to S18, >50% of J5 got hydrolyzed in just 6 hours under identical conditions (Figure 4). Further, proteolytic stability of J5 or S18 was also analyzed in normal mouse serum, a system which probably mimics *in-vivo* conditions. Fluorescein isothiocynate (FITC) conjugated analogs were incubated in serum at 37°C, aliquots were drawn at various time points and analyzed by gel electrophoresis. In this case, S18 could be visualized upto 24 hours, while 90% of J5 disappeared in just 3 hours (Figure 4). Thus to conclude, incorporation of homo-β-amino acids drastically augments the peptidic stability.

### S18 ameliorates EAE

**Figure 5:**
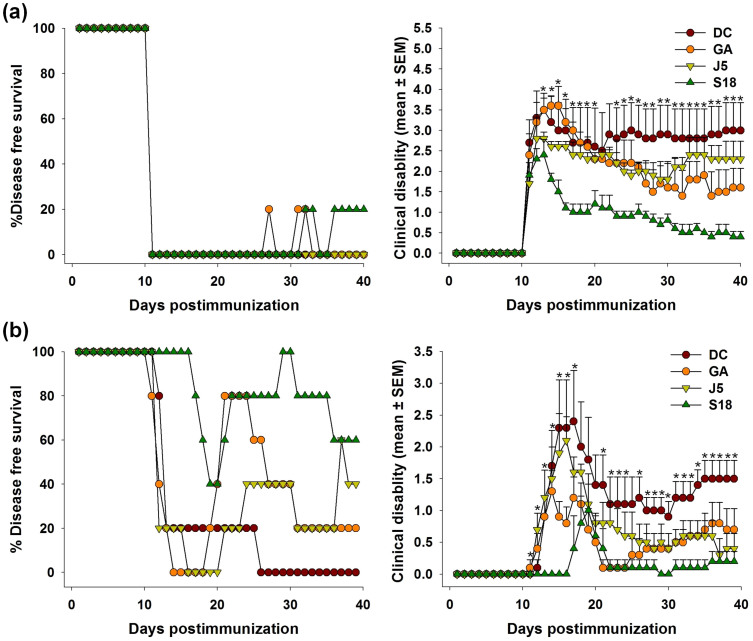
S18 ameliorates EAE. EAE was induced in *SJL/J* mice (8–10 week old, female) by immunization with MBP(85–99). Diseased animals were treated with 100 μg of GA, J5, S18 or vehicle (PBS) daily for 1 week, (a) percent disease-free survival and clinical disability score. *SJL/J* mice (8–10 week old, female) were pre-immunized with 400 μg of GA, J5, S18 or vehicle (PBS) in incomplete Freund's adjuvant (IFA) a week prior to immunization with MBP(85–99), (b) percent disease-free survival and clinical disability score. Mean ± SEM, *p<0.05, n ≥ 3, where n is number of independent experiments.

**Figure 6:**
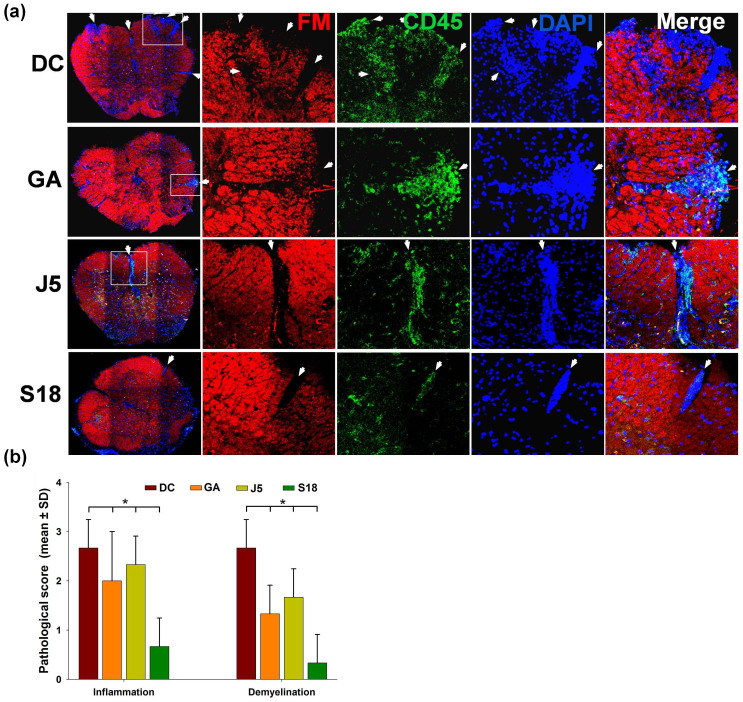
S18 ameliorates EAE: Immunohistochemical analysis. Cryosections of spinal cord (lumbar region) derived from S18, J5, GA or vehicle treated (disease control; DC) EAE mice at the end of 4 weeks were immunostained with anti-CD45 and counter-stained with fluoromyelin (FM, myelin stain), (a) photomicrographs at magnification of 10X, (b) histopathological score. Mean ± SD., *p<0.05, n ≥ 3, where n is number of independent cryosections analyzed.

Autoimmune demyelinating disorder with characteristics similar to MS was induced in experimental *SJL/J* mice by immunization with MBP(85–99). Approximately 75% of mice immunized with MBP(85–99) exhibited paralytic symptoms on day 11. The severity of disease peaked (mean clinical score 3.5) around day 13 with a mortality rate of 5–10 percent. The therapeutic efficacy of S18 was tested in two different scenarios i.e. treatment and prevention. In the former case, diseased animals were randomized into various experimental groups on appearance of clinical signs and treated with S18, J5, GA (100 μg, subcutaneously) or vehicle (PBS) daily for a week. Unlike, vehicle treated group (disease control), clinical disability in S18 treated animals did not progress beyond a mean score of 2.4. Moreover, the disease severity (measured in terms of cumulative disability score i.e. summation of clinical disability scores observed over the entire experimental duration) was reduced by ~65% on treatment with analog S18 while in the case of GA or J5 therapeutic remission was only 30–35% (Table S2). Comparatively, therapeutic effect of S18 treatment lasted for a longer duration (~3.2 weeks post-treatment) than J5 or GA where it could be seen only for ~1.7 weeks post treatment (Figure 5a). A relatively sustained therapeutic effect observed is expectedly an outcome of enhanced bioavailability of S18. For dosage response analysis, animals exhibiting EAE were treated with varying amounts of S18. Initially, the therapeutic response increased proportionately with dosage and saturated thereafter. A daily dosage of 100 μg was found to be optimum (Supplementary Fig. S3). In the prevention setup, experimental animals were injected with 400 μg of S18, J5, GA or vehicle (PBS) a week prior to immunization with MBP(85–99). In comparison to treatment experiment the mean clinical disability score of various pre-treatment groups (including disease control) was lower which essentially reflects disease-free survival in 20–30% of MBP(85–99) immunized animals. The disease severity (cumulative disability score) was reduced by ~80% in mice injected with S18 whereas GA or J5 treatment resulted in ~40–50% amelioration (Table S2). In addition to symptomatic relief, a delay of ~2 weeks was also noted in clinical onset of disease (Figure 5b). The delayed onset in this case may also be attributed to improved bioavailability of S18. Besides, gross symptomatic analysis, the effect of S18 treatment was also evaluated histopathologically. For this spinal cord tissue of treated or control animals was dissected out at the end of 4 weeks. Since, lower one third of spinal cord is primarily affected; cryosections derived from lumbar region were assessed for infiltration by mononuclear cells (inflammation) and myelin loss (demyelination). The degree of inflammation and resultant demyelination was analyzed by immunostaining with anti-CD45 and counterstaining with fluoromyelin. Fluoromyelin is a fluorescent dye that specifically stains the myelin component. The cryosections derived from vehicle treated (DC) mice displayed extensive inflammation and demyelination (histopathologic score 2.5–3.0) which was found to reduce significantly upon treatment with S18 (histopathologic score 0–0.5) (Figure 6a–b). In summary newly designed MBP(85–99) analog S18 ameliorates EAE with efficacy ~2 fold greater than that of GA or J5.

### Co-immunization with S18 and MBP(85–99) down-modulates recall response

**Figure 7:**
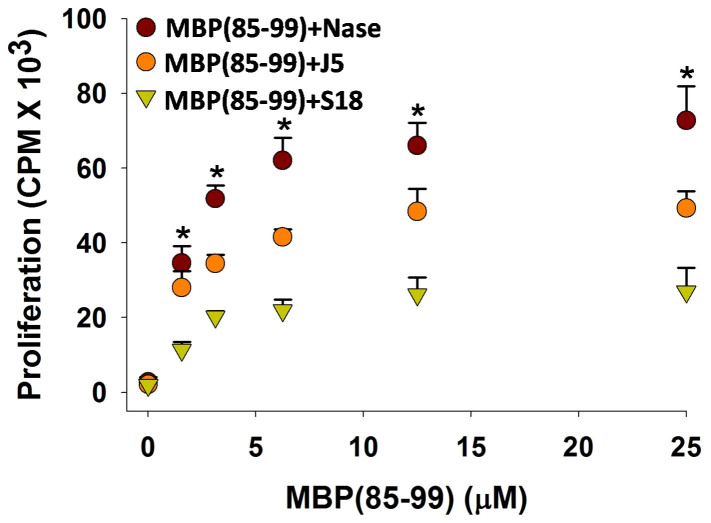
Co-immunization with S18 down-modulates recall response. *SJL/J* mice (8–10 week old, female) were co-immunized with 100 μg of MBP(85–99) and 400 μg of Nase(101–120), J5 or S18 in CFA. Proliferative activity of splenocytes isolated 2 weeks post-immunization in response to MBP(85–99). Mean ± S.D., *p<0.05, n ≥ 3, where n is number of independent experiments.

To investigate the effect of S18 treatment on generation of MBP(85–99) specific immune-reactivities *in-situ, SJL/J* mice were co-immunized with MBP(85–99) (100 μg) and 400 μg of S18, J5 or control peptide (Nase(101–120), unrelated to etiology of MS) in CFA. Two weeks post-immunization CD4^+^ T-cells derived from spleen were examined for proliferative response to MBP(85–99). Expectedly, splenocytes derived from control group responded vigorously to MBP(85–99) while proliferative response was markedly reduced in the case of animals co-immunized with S18 (Figure 7). As recall response to MBP(85–99) is a direct measure of frequencies of MBP(85–99) reactive CD4^+^ T-cells in the immunized animals, the observation thus indicates a marked decrease in frequencies of MBP(85–99) reactive CD4^+^ T-cells in animals treated with S18.

### S18 reactive CD4^+^ T-cell clones had protective effect

**Figure 8:**
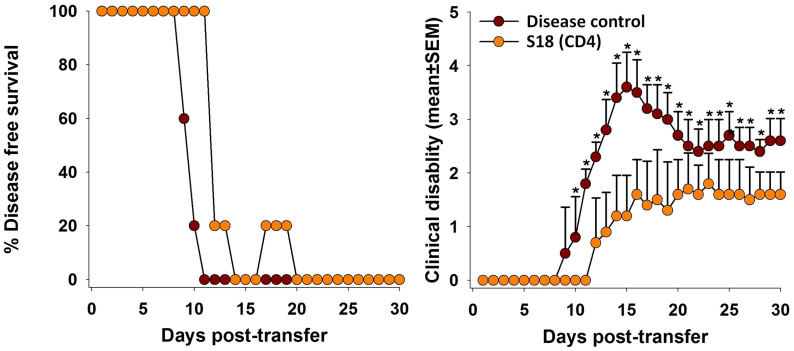
S18 reactive T-cell clones are protective. *SJL/J* mice (6–8 week old, female) prior to immunization with MBP (85–99) were adoptively transferred with S18 reactive CD4^+^ T-cell clones generated *in-vitro* (20 million). Percent disease-free survival and clinical disability score. Mean ± SEM, *p<0.05, n ≥ 3, where n is number of independent experiments.

The observations thus far suggest two mechanisms through which S18 might exert its therapeutic effect. First, by blocking MBP(85–99) presentation, hence, priming of auto-reactive CD4^+^ T-cells; secondly, by generating S18 reactive CD4^+^ T-cells with a suppressive phenotype, observed *in-vitro* (Figure 3b–c). To substantiate the latter case further, S18 reactive CD4^+^ T-cells generated *in-vitro* (as described in experimental section) were adoptively transferred into *SJL/J* mice a day prior to disease induction. Approximately ~50% mitigation of clinical symptoms with a marginal delay of 2 to 3 days in onset of disease was readily evident (Figure 8; Supplementary Table S2). Thus, positive outcome of adoptive transfer also demonstrates potential therapeutic activity of S18 reactive suppressive CD4^+^ T cells.

## Discussion

Stability in proteolytic environments is critical to biological activity of therapeutic peptides. There are various strategies that have been employed previously to enhance their stabilities *in-vivo*. The most popular of them are incorporation of unnatural amino acids, side chain fluorination, cyclization, etc.^28^,^29^,^30^,^31^. Among various unnatural amino acids usage of β or those with D conformations are quite common. However, the usage of β-amino acids for improving immuno-modulatory properties of therapeutic peptides is largely un-explored.

In the present study incorporation β-amino acids particularly homo-β were preferred because of their three very important characteristics, one, extreme resistance to proteases, two, flexibility of peptide backbone and third, ability to cross cellular barriers^27^,^32^. Homo-β-amino acids have their amino group attached to β-carbon instead of α-carbon and side chains (R groups) identical to that of their naturally occurring counterparts. Substitutions of α-amino acids with β elongate the peptide backbone by one carbon (CH2) with each residue incorporated. Consequently, the bulky groups attached to α and β carbons in a β or α-β hybrid peptide exhibit relatively high rotational freedom around them, thereby making them a lot more flexible and thermodynamically stable^32^.

In diseases associated with anomalous antigen presentation such as MS it is essential that therapies correcting the functional abnormality be stable enough. We preferred J5 over MBP(85–99) as starting material since it was the best known antagonist of MBP(85–99) evolved over a period of time and secondly, we believed that its therapeutic efficacy could be significantly enhanced by improving its biological half-life. Homo-β-amino acid substitutions in J5 were carried out considering the key features of MBP(85–99) or J5 and HLA-DR2b or TCR interactions. The substitutions in C-terminal region (P6 to P11) had no effect on inhibitory potentials which could be explained as this stretch of amino acids is considered to be less important for interaction with HLA-DR2b^12^,^26^. The deleterious effect on antagonistic activity observed upon single substitutions in the region extending from P1 to P4, the structural motif critical for interaction with HLA-DR2b, might be a result of certain conformational changes such as elongation of peptide backbone and drifting apart of major MHC anchors (side chains) i.e. valine (V) and tyrosine (Y) at P1 and P4. Unlike C-terminus, N-terminus of MBP(85–99) is deep seated in peptide binding groove of HLA-DR2b^12^, thus the deleterious effect of substitutions in this region (P-4 to P-1) observed could be explained as residues at these positions might be collectively contributing to binding affinity. Further, length of peptide backbone appeared to govern inhibitory activity as observed in the case of analogs S17 and S18. Analog S18 with its backbone length comparable to J5 was antagonistically more potent than its elongated counterpart S17 suggestive of a threshold that in this case might exist for the length of the peptide which HLA-DR2b molecule could comfortably accommodate in its peptide binding groove. However, further reductions drastically decreased the inhibitory activity attributable to the loss of binding motif itself.

Recognition of MBP(85–99)-HLA-DR2b complex by their cognate TCR, for example Ob.1A12, is central to activation of auto-reactive T cells and pathogenesis of MS. Although S18 could bind to HLA-DR2b but was not recognized by Ob.1A12 TCR present on MBP(85–99) reactive CD4^+^ T-cells as evident from *in-vitro* and *in-vivo* assays. Instead the S18-HLA-DR2b complex generates new specificities which we call as “S18-reactive CD4^+^ T cell clones”. These S18 reactive CD4^+^ T-cell clones were predominantly Th2 type, IL-4 secreting. The generation of Th2 phenotype might be an outcome of sub-optimal signaling through TCR. The prophylactic effect of S18 observed in pre-treatment setup is partly S18-reactive CD4^+^ T-cell mediated which could be further confirmed by protection they conferred upon their infusion into animals with EAE. Extended therapeutic benefit in treated or pre-treated animals could be explained on the basis of persistence of homo-β-amino acid containing S18. Alternatively, in the above scenario, auto-reactivities were either not initiated or their further generation was inhibited, which is an incessant phenomenon.

The designed analogs provided superior therapeutic benefits when compared to J5. The effect was noted to persist for more than 3 weeks in the treated animals after stopping the treatment which may be attributed to their longer *in-vivo* half-lives as deduced from their remarkable resistance towards proteolysis. On the contrary, J5 failed to confer extended therapeutic benefits due to rapid degradation post treatment. Same is reflected in the pre-treatment setup in terms of delayed onset in analog-treated mice.

Classical EAE initially manifests as immune cell infiltration and demyelination in the lower half of the spinal cord which ascends upwards with disease progression. The motor dysfunction in EAE is principally an outcome of immunological insult to the neural circuitries co-ordinating motor functions. Unaffected motor activities in analog treated mice were determined to be an outcome of reduced infiltration of spinal cord in the lumbar region by mononuclear cells.

Thus, in the present study stable homo-β-amino acid containing analogs of MBP (85–99) were much more effective in suppressing the presentation of MBP (85–99) to auto-reactive T-cells subsequently down-modulating their activation and ultimately getting translated into mitigation of disease symptoms in a mouse model of MS.

## Methods

### Mice

*SJL/J* mice (female; 6–8 week old) procured from Jackson Laboratories, were bred and housed under standard housing conditions. The mice were acclimatized for at least one week before the start of experimental procedures. All animals received *ad-libitum* access to food and water. Experimental procedures carried out on mice had prior approval of Institutional Animal Ethics Committee (IAEC) of *National Institute of Immunology*. Further, all methods were carried out in accordance with approved guidelines.

### Experimental Autoimmune Encephalomyelitis (EAE) induction and assessment

EAE was induced and assessed as described previously^33^. *SJL/J* (female; 6–8 week old) were injected subcutaneously with 100 μg of MBP(85–99) (two immunizations spaced a week apart) emulsified in complete Freund's adjuvant (CFA). Immunized mice were also given an intra-peritoneal injection of pertussis toxin (200 ng) on day 0 and 2. Severity of clinical disability was scored on a scale of 0 to 5, where, 0, no detectable signs of EAE; 1, complete tail paralysis; 2, wobbly gait; 3, complete hind limb paralysis; 4, complete hind and fore limb paralysis or moribund; 5, dead.

### Peptides, antibodies, cell lines and media

Peptides used namely MBP(85–99), Biotin-SGSG-MBP(85–99), Nase(101–120), J5, FITC-J5, S1–S20, FITC-S18 were atleast 95% pure (Bioconcept, Biocon, Genpro Biotech). Glatiramer acetate (Copaxone) was procured from Teva Pharmaceuticals (Israel). For treatment, peptides were freshly reconstituted in PBS. Antibodies, anti-HLA-DR (L243), anti-CD45, anti-CD4, anti-IFNγ, anti-IL-4, were purchased from BD Pharmingen and Abcam. Soluble HLA-DR2b-CLIP (HLA-DR2b having its trans-membrane region replaced with Fos-Jun dimerization motifs and a CLIP fragment attached through a thrombin cleavable linker to N-terminus of its β chain^34^,^35^), MGAR (EBV transformed B-cell line homozygous for HLA-DRB1*1501^36^ and Ob.1A12 (an MS patient derived auto-reactive CD4^+^ T-cell clone which recognizes MBP(85–99) in the context of HLA-DR2b^26^ were a kind gift from Dr. Kai W. Wucherpfennig (Dana-Farber Cancer Institute, USA). Cell lines mentioned above were cultured as per supplier's instructions. Tissue culture media Dulbecco's Modified Eagle Medium (DMEM), RPMI-1640, heat inactivated fetal bovine serum (FBS), human serum (HS), GlutaMax, antibiotic-antimycotic solution (Anti-anti) were purchased from Gibco (USA).

### Generation of S18 reactive T cell clones and adoptive transfer

*SJL/J* mice (6–8 weeks old, female) were immunized subcutaneously with 100 μg of S18 emulsified in CFA. At the end of 2 weeks splenocytes were harvested and co-cultured with stimulators at a ratio of 1:1. Stimulators were prepared by loading healthy mice derived irradiated splenocytes with S18 (10 μg/ml). Co-cultures were plated (5 million cells/ml) in primary culture medium (DMEM supplemented with 10% FBS, 2 mM GlutaMax, Anti-anti, 0.5 μM β-mercaptoethanol and 10 U/ml of recombinant human IL-2 (Sigma)). Cultures were replenished with IL-2 every 3–4 days and re-stimulated weekly using stimulators. At the end of 2 weeks S18 reactive CD4^+^ T-cells were isolated from mixed culture by magnetic sorting (Miltenyi Biotec) and infused (20 million cells/mice) a day prior to EAE induction.

### Intracellular staining and FACS analysis

For flowcytometeric analysis cells were harvested, washed, resuspended in staining buffer (BD). To block non-specific binding, cells were incubated with Fc-block (1 μg/million cells, BD) for 15 minutes on ice. Thereafter cells were washed and incubated with PE-anti-CD4 for 30 minutes on ice. Further for intracellular staining, cells were washed and resuspended in cytofix/cytoperm buffer (BD) for 15 minutes at room temperature, washed with perm/wash buffer (BD) and incubated with FITC-anti-IL-4 or APC-anti-IFNγ for 30 minutes on ice. On completion of incubation period cells were washed twice with perm/wash buffer and resuspended in staining buffer. Data was acquired on BD FACS Calibur and analyzed using Flowing Software 2.5.1.

### Cryosectioning and Immunohistochemistry

Animals were perfused intra-cardially with ice cold PBS and 4% paraformaldehyde (PFA) sequentially under deep anesthesia. Thereafter intact spinal cord tissue was dissected out, equilibrated with 30% sucrose and embedded in tissue freezing media (Polyfreez, Polysciences). Subsequently, transverse sections (15 μM thick) were cut through lumbar region in a cryotome (Thermo) and collected in storage buffer (30% glycerol and ethylene glycol in PBS). Sections were retrieved from storage buffer, transferred into citrate buffer (10 mM, pH 6.0) and incubated at 65°C for 30 minutes for antigen retrieval. After 30 minute incubation period, sections were equilibrated with PBS and blocked using 3% bovine serum albumin and 1% normal serum for 2 hours at room temperature. Thereafter sections were incubated with primary antibody (anti-CD45) overnight at 4°C, washed with PBST and incubated with secondary antibody (Alexa 488-anti-rabbit) for 2 hours at room temperature. After washing with PBST, sections were counter-stained with Fluoromyelin (Invitrogen), a myelin stain (1:600 dilution in PBS at room temperature for 15 minutes). Finally, stained sections were washed with PBST, transferred onto glass slides pre-coated with silane (Silane-prep slides, Sigma) and mounted with fluoroshield-DAPI (Sigma). Images were acquired in confocal microscope equipped with motorized stage (Leica TCS SP8) at 10X magnifications and processed using LAS AF Lite software. Histopathologic scoring depicting extent of mononuclear infiltration (inflammation) and demyelination was carried out on a scale of 0–3 where, 0 indicated no inflammation/demyelination, 1- mild inflammation/demyelination, 2- moderate inflammation/demyelination, 3- severe inflammation/demyelination.

### Peptide binding assay

Before setting peptide exchange reactions HLA-DR2b-CLIP was treated with thrombin (Sigma) at a concentration of 20 U/mg for 2 h at room temperature so as to facilitate the release of CLIP peptide from peptide binding groove. Thrombin treated HLA-DR2b-CLIP (0.2 μM) was incubated with biotin-MBP(85–99) (2 μM) and/or scrambled MBP(85–99), MBP(85–99), J5, S1 to S20 at various molar ratios for 24 h at 37°C in 50 μL of the binding buffer (DPBS, 1 mM EDTA, 1 mM PMSF, pH 7.2). The amount of biotin-MBP(85–99) bound to HLA-DR2b was estimated by ELISA. The reaction mixtures were transferred into anti-HLA-DR (L243) coated 96 well microtiter assay plates and incubated at 37°C for 1 h. Coating with anti-HLA-DR (1 μg/well) was done in 100 μL carbonate-bicarbonate buffer (0.1 M, pH 8.0) overnight at 4°C. Biotin-MBP(85–99) bound to HLA-DR2b was detected using streptavidin-HRP (BD). 3, 3′, 5, 5′-tetramethylbenzidine (TMB) was added to each well and absorbance at 410 nm was recorded on an ELISA reader (TECAN infinite M200). Percent inhibition was calculated with respect to blank i.e. when HLA-DR2b were incubated with biotin-MBP(85–99) alone.

### Cell surface MBP(85–99) binding assay

Antagonistic activities of MBP(85–99) analogs were assayed as described previously^37^,^38^. Briefly, MGAR cells (0.1 million) growing in logarithmic phase were harvested, irradiated (150 Grey) and incubated with biotin-MBP(85–99) (1.5 μM) and/or scrambled MBP(85–99), J5 or S18 (1.5 μM, molar ratio 1:1; 15 μM, molar ratio of 1:10; 150 μM, molar ratio 1:100) in FACS staining buffer (BD) for 2 h at room temperature. Subsequently, cells were washed and surface bound biotin-MBP(85–99) molecules were detected using streptavidin-APC (30 minute incubation at 4°C). Thereafter, cells were washed and acquired on BD FACS Calibur and analyzed using Flowing Software 2.5.1. Binding of MBP(85–99) to cell surface HLA-DR2b was measured in terms of mean fluorescence intensity (MFI) and percent inhibition was calculated with respect to control i.e. when irradiated MGAR cells were incubated with Biotin-MBP(85–99) alone.

### *In-vitro* MBP(85–99) presentation assay

MGAR cells growing in logarithmic phase were irradiated (150 Grey). Irradiated MGAR cells (0.1 million) were pulsed with MBP(85–99) (1.5 μM) and/or scrambled MBP(85–99), J5 or S18 at various molar ratios (1:1, 1:10, 1:100) and co-cultured with Ob.1A12 cells (0.1 million). To determine proliferative activity of Ob.1A12, cultures were pulsed with 0.5 μCi of [^3^H] thymidine (Perkinelmer) at the end 48 hours, after 16 hours harvested onto Filtermat A (Wallac) and read in a plate scintillation counter (Perkinelmer). Also, levels of IL-2 were measured by ELISA at the end of 48 hours.

### Proteolysis assay

MBP(85–99) analogs J5 or S18 (>95% pure, 1 mg/ml) were incubated with chymotrypsin (0.1 μg/ml, ~4.0 BTEE Units/μg) in assay buffer (PBS containing 0.01% sodium azide) at 37°C. Aliquots were drawn at various time points and analyzed by high pressure liquid chromatography (HPLC, Shimadzu). Degradation mixtures were resolved on a C18 column (Phenomenix) using acetonitrile/water with 0.1% trifluroacetic acid (TFA) as a solvent system at a flow rate of 0.4 ml/minute. Analogs un-cleaved in the reaction mixture were quantified in terms of percent peak area. Also, analogs J5 or S18 labeled with FITC at N-terminus were incubated in 10% normal mouse serum (diluted in assay buffer) at 37°C. Aliquots were drawn and analyzed on a polyacrylamide gel (15%).

### Cytokine Assay

The levels of IFNγ, IL-2, IL-4 and IL-10 in culture supernatants were quantified using Ready set go ELISA kit (Ebioscience) following manufacturer's instruction. Values observed were normalized to control (media).

### In-silico analysis

Docking studies were performed using Molecular Operating Environment version 2013.0802. *Preparation of receptor and ligands:* HLA-DR2b crystal structure submitted to Protein Data Bank (www.rcsb.org, PDB ID: 1BX2) was used as a receptor in the docking experiment, particularly, chain A and B^12^. Ligand molecules were built using Chemdraw Ultra 7.0 in skc file format. The receptor and ligand molecules were optimized for docking experiment employing LigX application of MOE. The process of receptor or ligand preparation involved fixation of partial charges, protonation and energy minimization (dielectric constant 1.0, energy minimization gradient 0.0001 and force field MMFF94x).

*Docking:* The optimized ligand molecules compiled into a database file were docked into the active site (peptide binding groove) of receptor molecule using default non-stochastic triangular matcher replacement method with London G scoring followed by forcefield refinement.

### Data analysis

Student's t-test (unpaired, two tailed) and analysis of variance (ANOVA, single factor) were employed for binary and multiple comparisons respectively. Differences in mean values were considered significant when p < 0.05.

## Author Contributions

A.S. and R.K. conceived and designed the research. R.K. and S.P. performed the experiments. A.S., R.K. and S.P. analyzed data and wrote the manuscript.

## Supplementary Material

Supplementary Information Supplementary information
