# Supplementary material for: Homo-β-amino acid containing MBP(85–99) analogs alleviate experimental autoimmune encephalomyelitis
Source: Sci Rep. 2015 Feb 3;5:8205. doi: 10.1038/srep08205 (PMC4314633; doi:10.1038/srep08205)
Supplement: Supplementary Information [file srep08205-s1.pdf]

Supplementary data

Homo-β-amino acid containing MBP(85-99) analogs alleviate experimental autoimmune encephalomyelitis

Ravi Kant[1], Shweta Pasi[1], Avadhesha Surolia[1, 2, *]

[1]Molecular Science Laboratory, National Institute of Immunology, New Delhi-110067, India.

[2]Present Address: Molecular Biophysics Unit, Indian Institute of Science, Bangalore-560012, India.

[*]Corresponding author e-mail address: surolia@mbu.iisc.ernet.in

.

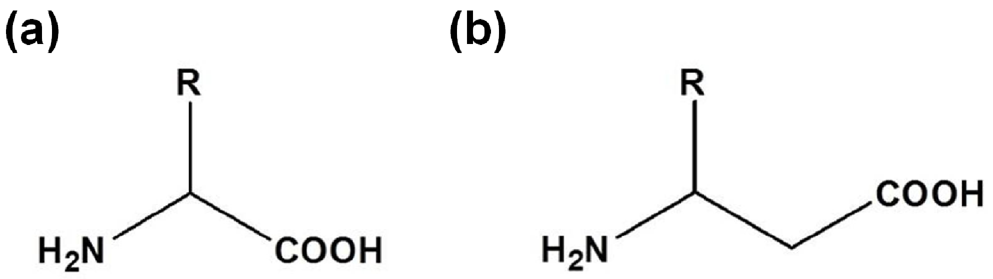

(c)

	P1 P2 P3 P4 P5
MBP(85-99)	ENPVVHFFKNIVPTR
J5	EKPKVEAYKAAAAPA

Figure S1. MBP(85-99), J5 comparison. (a) α-amino acid. **(b)** Homo - β - amino acid. **(c)** Pair-wise aligned sequences of MBP(85-99) and J5. Major MHC anchors i.e. valine (V) and phenylalanine (F); their positions P1 and P4 are marked in red. TCR contacts i.e. histidine (H), phenylalanine (F) and lysine (K); their positions P2, P3 and P5 are marked in purple.

ID	SEQUENCE	%INHIBITION ± SD
MBP(85-99)	ENPVVHFFKNIVPTR	37.1 ± 3.2
J5	EKPKVEAYKAAAAPA	43.3 ± 4.3
S1	EKPKVEAYKAAAAPA_{β}^{3}	42.0 ± 1.7
S2	EKPKVEAYKAAAAP_{β}^{3}A	40.6 ± 4.4
S3	EKPKVEAYKAAAA_{β}^{3}PA	44.4 ± 2.5
S4	EKPKVEAYKAAA_{β}^{3}APA	41.7 ± 4.3
S5	EKPKVEAYKAA_{β}^{3}AAPA	39.9 ± 1.5
S6	EKPKVEAYKA_{β}^{3}AAAPA	45.5 ± 3.5
S7	EKPKVEAYK_{β}^{3}AAAAPA	42.6 ± 4.1
S8	EKPKVEAY_{β}^{3}KAAAAPA	31.4 ± 2.4*
S9	EKPKVEA_{β}^{3}YKAAAAPA	28.1 ± 4.3*
S10	EKPKVE_{β}^{3}AYKAAAAPA	26.3 ± 3.9*
S11	EKPKV_{β}^{3}EAYKAAAAPA	22.4 ± 8.9*
S12	EKPKVEAY$K_{\beta}^{3}A_{\beta}^{3}A_{\beta}^{3}A_{\beta}^{3}A_{\beta}^{3}P_{\beta}^{3}A_{\beta}^{3}$	40.1 ± 6.1
S13	EKPK_{β}^{3}VEAY$K_{\beta}^{3}A_{\beta}^{3}A_{\beta}^{3}A_{\beta}^{3}A_{\beta}^{3}P_{\beta}^{3}A_{\beta}^{3}$	34.4 ± 2.4*
S14	EKP_{β}^{3}KVEAY$K_{\beta}^{3}A_{\beta}^{3}A_{\beta}^{3}A_{\beta}^{3}A_{\beta}^{3}P_{\beta}^{3}A_{\beta}^{3}$	33.0 ± 3.6
S15	EK_{β}^{3}PKVEAY$K_{\beta}^{3}A_{\beta}^{3}A_{\beta}^{3}A_{\beta}^{3}A_{\beta}^{3}P_{\beta}^{3}A_{\beta}^{3}$	39.8 ± 4.9
S16	E_{β}^{3}KPKVEAY$K_{\beta}^{3}A_{\beta}^{3}A_{\beta}^{3}A_{\beta}^{3}A_{\beta}^{3}P_{\beta}^{3}A_{\beta}^{3}$	40.3 ± 5.2
S17	$E_{\beta}^{3}K_{\beta}^{3}$PKVEAY$K_{\beta}^{3}A_{\beta}^{3}A_{\beta}^{3}A_{\beta}^{3}A_{\beta}^{3}P_{\beta}^{3}A_{\beta}^{3}$	40.1 ± 5.7
S18	$E_{\beta}^{3}K_{\beta}^{3}$PKVEAY$K_{\beta}^{3}A_{\beta}^{3}A_{\beta}^{3}A_{\beta}^{3}A_{\beta}^{3}$	59.5 ± 8.6*
S19	VEAY$K_{\beta}^{3}A_{\beta}^{3}A_{\beta}^{3}A_{\beta}^{3}A_{\beta}^{3}$	14.0 ± 2.9*
S20	VEAYK_{β}^{3}	16.5 ± 5.1*

*p<0.05, student's t-test (unpaired, two tailed), all comparisons with respect to J5 (control).

Table S1. Percent antagonistic activities of homo-β-amino acid substituted MBP(85-99) analogs at 1:10 molar ratio i.e 0.5µM biotin-MBP(85-99) and 5 µM analog.

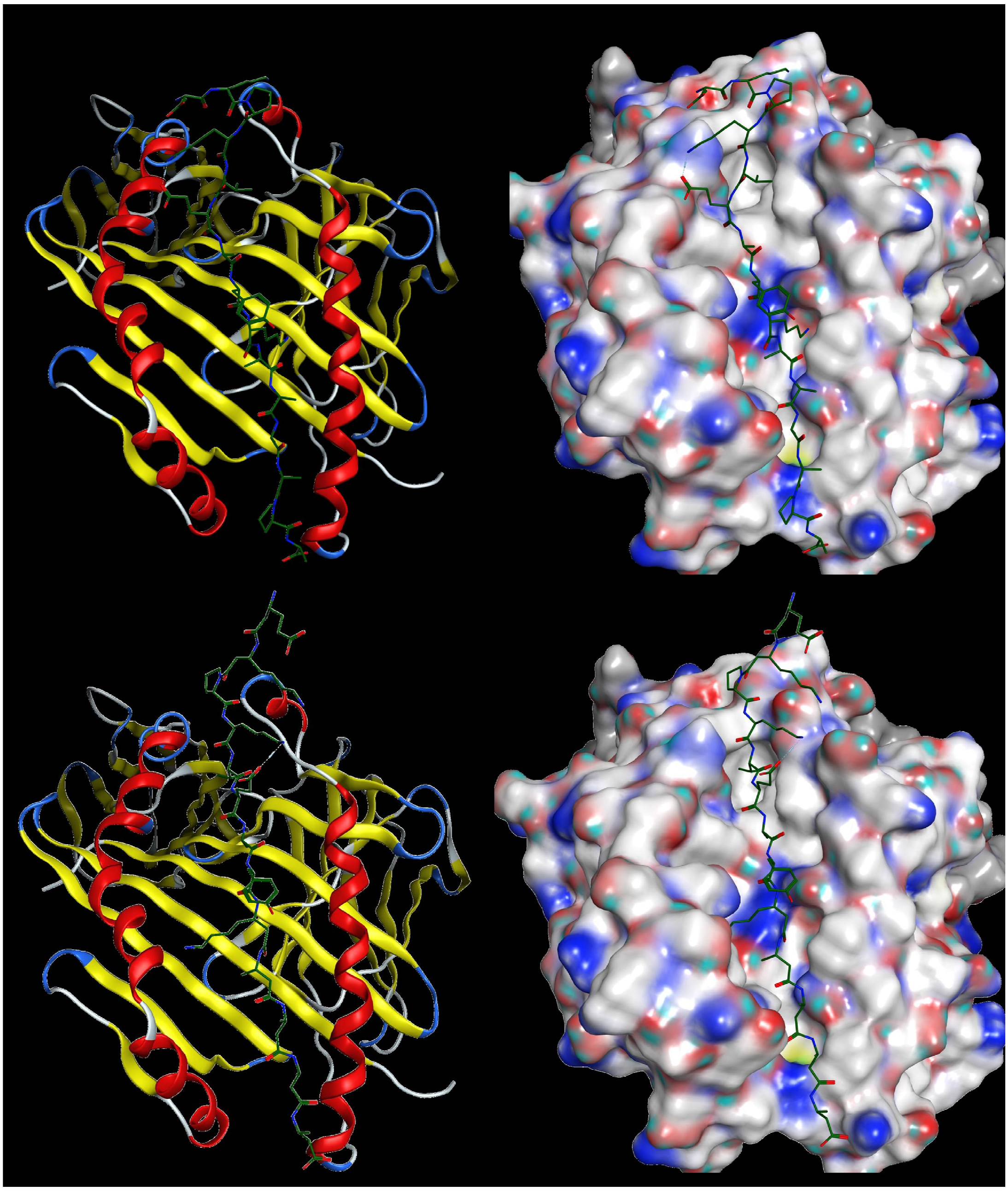

J5

S18

Figure S2. In-silico analysis. Ligands J5 or S18 were docked onto HLA-DR2b, bound conformations as predicted by MOE 2013.0802.

Experiment	Treatment group	Mean ± SEM
MBP(85-99) induced EAE : Treatment (cumulative disability score)	Disease control	86.6 ± 18.94
	GA	65.9 ± 11.32
	J5	67.8 ± 4.63
	S18	29.2 ± 3.17
$p < 0.05$, analysis of variance (one factor)		
MBP(85-99) induced EAE : Pre-treatment (cumulative disability score)	Disease control	38.3 ± 4.08
	GA	16.4 ± 4.23
	J5	22.9 ± 3.60
	S18	5.2 ± 1.83
$p < 0.05$, analysis of variance (one factor)		
MBP(85-99) induced EAE : Pre-treatment (clinical onset of disease)	Disease control	12.8 ± 0.26
	GA	12.2 ± 0.63
	J5	12.8 ± 1.03
	S18	24.8 ± 5.63
$p < 0.05$, analysis of variance (one factor)		
MBP(85-99) induced EAE : Adoptive transfer (clinical onset of disease)	Disease control	9.8 ± 0.48
	S18 (CD4)	12.6 ± 0.52
$p < 0.05$, analysis of variance (one factor)		
MBP(85-99) induced EAE : Adoptive transfer (cumulative disability score)	Disease control	55.9 ± 6.20
	S18 (CD4)	27.7 ± 7.32
$p < 0.05$, analysis of variance (one factor)		

Table S2. Treatment, Pre-treatment and passive transfer: cumulative disability score (total clinical disability score recorded over the entire experimental period) and clinical onset of EAE.

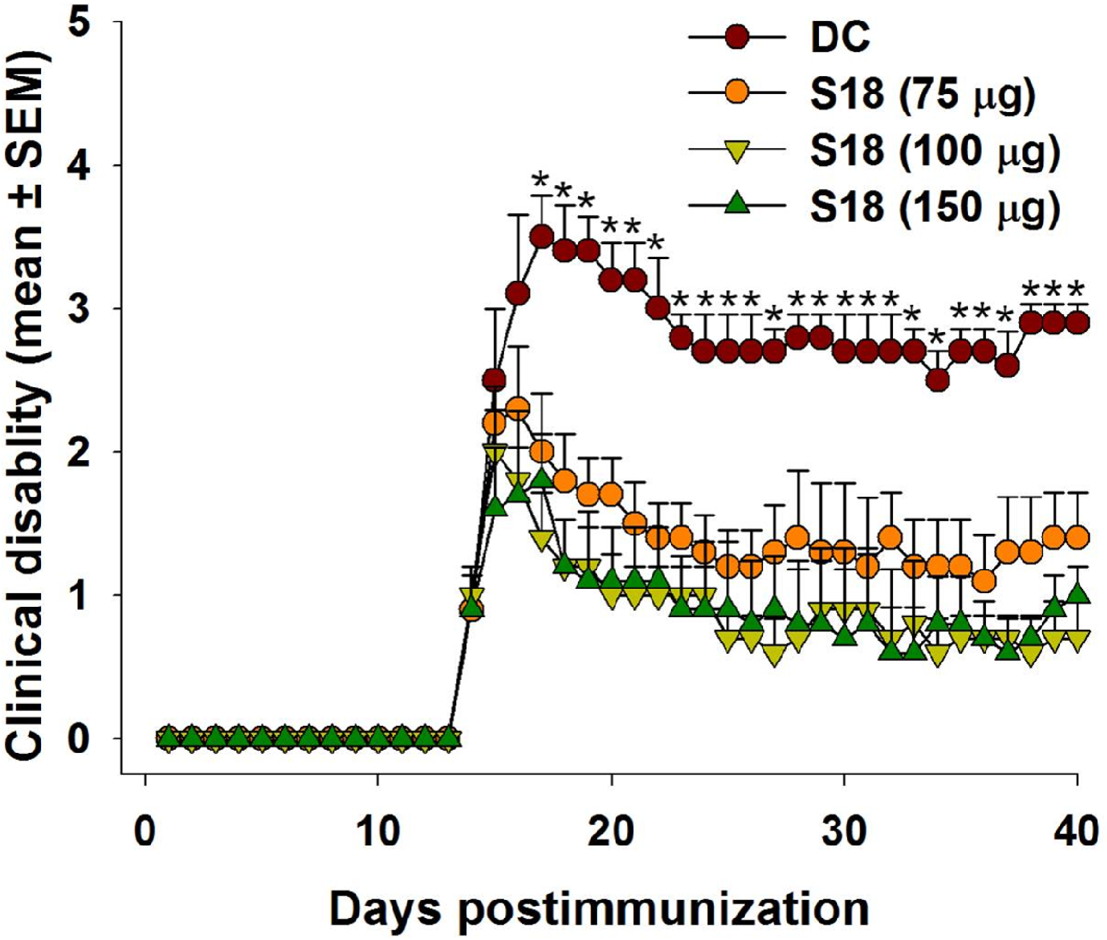

Figure S3. S18 treatment: dosage kinetics. EAE was induced in SJL/J mice (8-10 week old, female) by immunization with MBP(85-99). Diseased animals were treated with various dosages of S18 viz. 50 µg, 100 µg, 150 µg and 200 µg dailyfor 1 week. Clinical disability Score. Mean ± S.D., n≥3, where n is number of independent experiments.
